# Identification of phenylketonuria patient genotypes using single-gene full-length sequencing

**DOI:** 10.1186/s40246-022-00397-w

**Published:** 2022-07-22

**Authors:** Jinshuang Gao, Xiaole Li, Yaqing Guo, Haiyang Yu, Liying Song, Yang Fang, Erfeng Yuan, Qianqian Shi, Dehua Zhao, Enwu Yuan, Linlin Zhang

**Affiliations:** 1 The Department of Laboratory Medicine, Henan Key Laboratory of Child Brain Injury and Henan Pediatric Clinical Research Center, Third Affiliated Hospital and Institute of Neuroscience of Zhengzhou University, Zhengzhou, 450052 People’s Republic of China; 2 Neonatal Screening Center, The Third Affiliated Hospital of Zhengzhou University, Zhengzhou, 450052 People’s Republic of China

**Keywords:** Phenylketonuria, *PAH*, Single-gene full-length sequencing, Deep intronic variant

## Abstract

**Background:**

Phenylketonuria (PKU) is a common, autosomal recessive inborn error of metabolism caused by *PAH* gene variants. After routine genetic analysis methods were applied, approximately 5% of PKU patients were still not diagnosed with a definite genotype.

**Methods:**

In this study, for the first time, we identified PKU patients with unknown genotypes via single-gene full-length sequencing.

**Results:**

The detection rate of PKU genotype increased from 94.6 to 99.4%, an increase of approximately 5%. The variants c.1199 + 502A > T and 1065 + 241C > A were found at a high frequency in Chinese PKU patients.

**Conclusion:**

Our study suggest that single-gene full-length sequencing is a rapid, efficient and cost-effective tool to improve the genotype detection rate of PKU patients. Moreover, we provides additional case data to support pathogenicity of deep intronic variants in *PAH*.

**Supplementary Information:**

The online version contains supplementary material available at 10.1186/s40246-022-00397-w.

## Introduction

Phenylketonuria (PKU) is the most common congenital disorder of amino acid metabolism, with a global prevalence of 1:10,000 newborns [1]. PKU is due to a deficiency in phenylalanine hydroxylase (PAH) activity caused by the *PAH* variant [2]. When this enzyme is deficient, elevated phenylalanine (Phe) can have toxic effects on the nervous system. In line with the European guidelines [3] and Chinese guidelines [4] for PKU, it is classified into mild PKU (mPKU, 360–1200 μmol/L) and classic PKU (cPKU, ≥ 1200 μmol/L), depending on the Phe concentration in peripheral blood at the time of diagnosis. Many countries have included PKU in neonatal screening programs. According to the latest screening data, the incidence of PKU was 6.28:100,000 newborns in China from 2013 to 2017[5].

Genetic analysis is an effective means for early etiological diagnosis, treatment guidance and prenatal diagnosis of PKU patients. Based on Chinese guidelines [4] gene diagnosis is the diagnostic method of the etiology of HPA patients, and it is recommended to carry out routine testing, especially for patients with atypical biochemical phenotypes. Through *PAH* gene detection, PKU patients can be identified more quickly and effectively.

The American College of Medical Genetics and Genomics (ACMG) [6] suggests that mutation analysis should be obtained for all infants with elevated Phe. Because of the high prevalence rate of PKU, *PAH* has been included in carrier screening by ACMG [7].

At present, routine molecular genetic analysis is commonly used in the clinical detection of *PAH* variants, including Sanger sequencing, next-generation sequencing (NGS) gene panels and multiplex ligation-dependent probe amplification (MLPA). Through the analysis of the *PAH* gene variant spectrum in different populations, 87–96% of PKU patients can be identified by sequencing all exons and flanking intron regions [8–10]. Exonic deletion/duplication identified by MLPA accounts for 2–3% of pathogenic *PAH* alleles [11, 12]. Approximately 5% of the remaining patients have typical clinical symptoms, but the genotype is unknown [11].

An analysis of the genetic landscape of PKU found that among pathogenic variants of *PAH*, 17.9% occur in introns or untranslated regions [1]. However, although conventional molecular genetic analysis can detect these regions, it only contains a small number of known variants and cannot cover all deep introns, 5′UTR and 3′UTR regions. In this study, we applied full-length sequencing of *PAH* single genes in PKU patients with unknown genotypes and with deletion/duplication of *PAH*. We examined the use of single-gene full-length sequencing for characterizing the molecular genetics of PKU patients and provide additional reference data for variant detection of deep introns of *PAH*.

## Patients and methods

### Subjects

Based on clinical features and newborn screening, a total of 687 patients were diagnosed with PKU at the Third Affiliated Hospital of Zhengzhou University from January 2016 to December 2021. All of them underwent *PAH* gene molecular genetic analysis through the gene panel (including *PAH* and *DNAJC12*) by NGS and MLPA.

We reanalyzed the clinical phenotypes and molecular genetic results of these PKU families to screen out patients with unknown genotypes. The inclusion criteria were as follows: (1) exhibiting PKU phenotypes or increased blood Phe concentrations (≥ 360 μmol/L); (2) having only a single deleterious heterozygous variant or no variants identified after *PAH* gene molecular genetic analysis; (3) having been excluded from tetrahydrobiopterin deficiency.

**Fig. 1:**
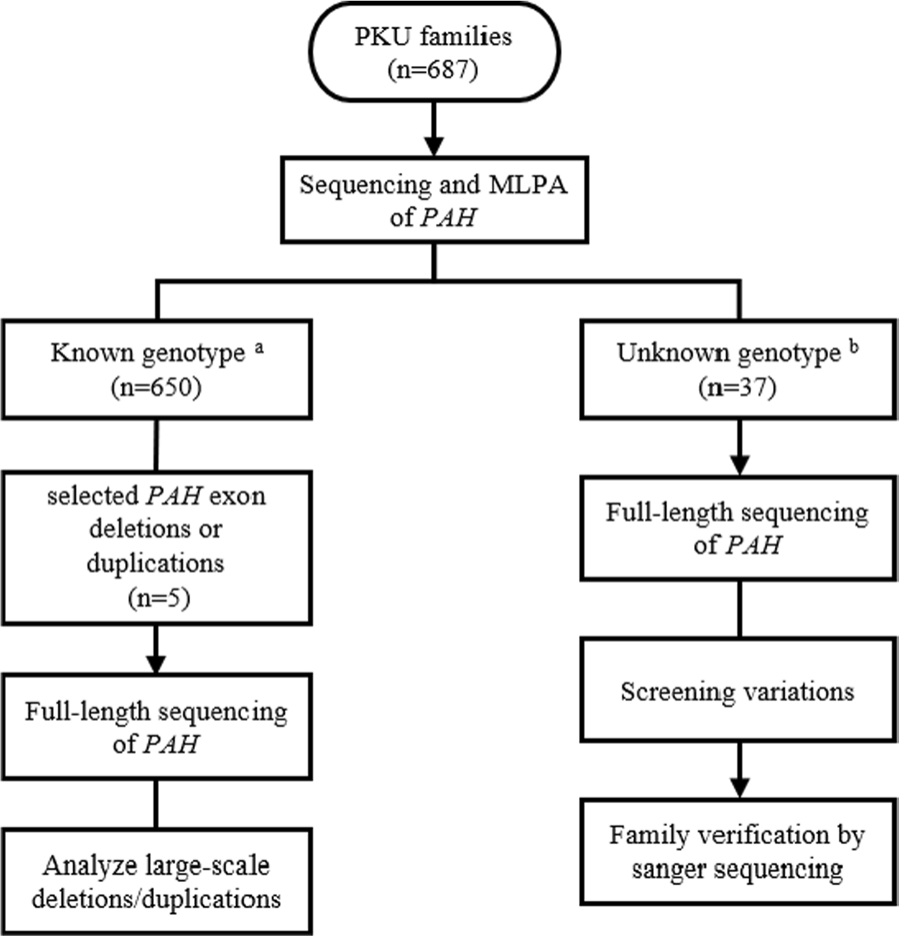
Research analysis workflow. a The patients were completely genotyped of which carried homozygous variant, compound heterozygous variants or three separate variants. b The patients identified only a single deleterious heterozygous variant or did not detect any variant

After screening, a total of 37 PKU patients with unknown genotypes and their family members were enrolled. Among them, thirty-six PKU patients had only a single deleterious heterozygous variant identified, and one patient did not have any variants detected. Samples from all of these probands underwent single-gene full-length sequencing. To test the detection efficiency of single-gene full-length sequencing, we selected 5 samples with different exon deletions or duplications in *PAH* detected by MLPA analysis for sequencing (Fig. 1). All PKU families signed informed consent, and the study passed the ethical approval of Third Affiliated Hospital of Zhengzhou University.

### Single-gene full-length sequencing

Sequencing of residual DNA specimens from the probands was used for library preparation and target capture. Library preparation included end repair, adapter ligation and PCR enrichment and was carried out as recommended by Illumina protocols. The amplified DNA was captured using a GenCap Phenylketonuria capture kit (MyGenostics GenCap Enrichment Technologies). An Illumina HiSeq X Ten Sequencer (Illumina, San Diego, USA) with 150 bp paired-end sequencing mode was used for sequencing the genomic DNA. The mean sequencing coverage was > 1000 × , and > 99.0% of bases for every sample were sequenced to at least 20 × coverage.

### Data analysis

The sequencing reads were aligned to the human reference genome (hg19/GRCh37) using BWA, and PCR duplicates were removed by using Picard v1.57 (http://picard.sourceforge.net/). The variants of single nucleotide variants (SNVs) and insertion-deletion (InDel) were detected by GATK (https://software.broadinstitute.org/gatk/). Variant annotation and interpretation were conducted by ANNOVAR [13] and associated with multiple databases. The annotation databases mainly included (1) human population databases, such as gnomAD (http://gnomad.broadinstitute.org/), the 1000 Genome Project (http://browser.1000genomes.org), “HUABIAO” whole-exome public database (https://www.biosino.org/wepd/index) and dbSNP (http://www.ncbi.nlm.nih.gov/snp); (2) in silico prediction algorithms, such as SIFT (http://sift.jcvi.org), FATHMM (http://fathmm.biocompute.org.uk), MutationAssessor (http://mutationassessor.org), and CADD (http://cadd.gs.washington.edu); and (3) disease and phenotype databases, such as ClinVar (http://www.ncbi.nlm.nih.gov/clinvar), HGMD (http://www.hgmd.org), and BIOPKU (http://www.biopku.org/home/home.asp). The impact of variants on the splice site was predicted by using Alamut® Visual Plus v1.1.

Large-scale deletion/duplication of *PAH* was identified using CNVkit [14]. Normal references used for CNV identification were obtained using sequencing data from 10 normal males and 10 females that had previously been validated without large deletions or duplications of *PAH* by MLPA.

### Variant validation and classification

All putatively causal variants were sequenced by Sanger method to confirm the genotypes of the probands and parents. The variants were classified into five categories: pathogenic, likely pathogenic, uncertain significance, likely benign and benign, according to the ClinGen *PAH* Expert Panel Specifications to the ACMG/AMP Variant Interpretation Guidelines [15].

### Analysis of nonbenign *PAH* variants in deep introns and untranslated regions

A total of 1282 variants were included in the PAHvdb database (as of June 18, 2021), and we screened the SNVs in deep introns and untranslated regions by manual screening. The screening criteria were as follows: (1) located on introns, 5′UTR or 3′UTR that distance exons > 20 bp; (2) having reliable supporting reports in PubMed (https://pubmed.ncbi.nlm.nih.gov/); (3) having no evidence of benign impact correlation in ClinVar and HGMD databases; (4) having an allele frequency no greater than 5% in the gnomAD database. We analyzed the variants screened manually and the deep intronic novel variants detected in patients in this study, including analyses of genome location and carrier population.

## Results

### SNV/indel detection

After screening, a total of 37 PKU patients with unknown genotypes were included. Their information, genotypic and phenotypic characteristics are summarized in Table 1.

**Table 1:** Genotypes and phenotypes of 37 PKU families

Patients	Age	Pre-treatment Phe levels (μmol/L)	Genotypes^a^	Classification
1	4y9d	606	c.[1065 + 241C > A];[208_210del]	mPKU
2	1m17d	2340	c.[1199 + 502A > T];[442-1G > A]	cPKU
3	5m20d	2622	c.[1030G > A];[1199 + 502A > T]	cPKU
4	1 m	1548	c.[740G > T];[1199 + 502A > T]	cPKU
5	7y	NA^b^	c.[1068C > A];[1199 + 502A > T]	cPKU
6	2m14d	540	c.[992 T > C];[?]	mPKU
7	6m12d	796	c.[1065 + 241C > A];[728G > A]	mPKU
8	3m5d	2099	c.[929C > T];[1199 + 502A > T]	cPKU
9	1m21d	696	c.[1199 + 502A > T];[722G > A]	mPKU
10	1m7d	3660	c.[331C > T];[1199 + 502A > T]	cPKU
11	2m2d	792	c.[707-59C > G];[1084C > A]	mPKU
12	17 m	1686	c.[1199 + 502A > T];[611A > G]	cPKU
13	1m17d	456	c.[1301C > A];[1065 + 241C > A]	mPKU
14	1m13d	900	c.[1199 + 502A > T];[1045 T > G]	mPKU
15	3y3m	2700	c.[1199 + 502A > T];[781C > T;1256A > G]	cPKU
16	1m6d	414	c.[1023G > C];[1065 + 241C > A]	mPKU
17	6y10m	936	c.[728G > A];[1065 + 241C > A]	mPKU
18	1m8d	828	c.[1162G > A];[?]	mPKU
19	2m5d	780	NA	mPKU
20	1m10d	840	c.[482 T > C];[1065 + 241C > A]	mPKU
21	26d	528	c.[707-59C > G];[208_210del]	mPKU
22	1m5d	1140	c.[1199 + 502A > T];[332G > A;1301C > A]	mPKU
23	30d	1494	c.[740G > T];[1199 + 502A > T]	cPKU
24	1m2d	720	c.[913-7A > G];[1199 + 502A > T]	mPKU
25	8y3m	1506	c.[1065 + 241C > A];[331C > T]	cPKU
26	1 m	1920	c.[**706 + 629A > C**];[526C > T]	cPKU
27	4m3d	2058	c.[158G > A;842 + 2 T > A];[1199 + 502A > T]	cPKU
28	1m20d	540	c.[1065 + 241C > A];[331C > T]	mPKU
29	1m9d	2274	c.[755G > A];[?]	cPKU
30	1m14d	1560	c.[611A > G];[1065 + 241C > A]	cPKU
31	21d	360	c.[251A > G];[1199 + 502A > T]	mPKU
32	1m2d	2160	c.[1199 + 502A > T];[158G > A;842 + 2 T > A]	cPKU
33	1y8m	1140	c.[728G > A];[1199 + 502A > T]	mPKU
34	1m6d	450	c.[284_286del];[1065 + 241C > A]	mPKU
35	16y	1860	c.[158G > A;842 + 2 T > A];[1199 + 502A > T]	cPKU
36	1m10d	2160	c.[1199 + 502A > T];[442-1G > A]	cPKU
37	2m11d	558	c.[728G > A];[1065 + 241C > A]	mPKU

The novel variant is shown in bold

NA: Not identified, cPKU: classic PKU(Phe ≥ 1,200 μmol/L), mPKU: mild PKU(Phe 360–1200 μmol/L)

^a^Reference sequence of variants is NM_000277.3

^b^Patient 5 was clinically diagnosed as classic PKU by musty odor from skin and urine, fair skin and intellectual disability

After single-gene full-length sequencing, 74 potential disease-causing variant alleles were identified. A total of 33 patients were completely genotyped, of which 28 carried compound heterozygous variants and 5 harbored three separate variants. Compared with the previous results, the detection rate of PKU increased from 94.6% (650/687) to 99.4% (683/687), an increase of approximately 5%. However, there were still four patients with unclear genotypes. Although they have identified some variants of uncertain significance, the pathogenicity of these variants may need more evidence (Additional file 1: Table S1).

Among the results from 33 patients identified by full-length sequencing, all of the newly detected variant alleles were in the deep introns, including c.707-59C > G, c.1065 + 241C > A, c.1199 + 502A > T and a novel variant c.706 + 629A > C (Table 2). The most frequent variant was c.1199 + 502A > T (57.6%), followed by c.1065 + 241C > A (33.3%) and c.707-59C > G (6.1%). It indicated that full-length sequencing could effectively detect the variants both of exons and deep introns.

**Table 2:** Deep intronic variants identified by full-length sequencing of *PAH*

Nucleotide aberration	Location	Frequency of detection in PKU patients	Proportion of mPKU	Proportion of cPKU	Variant classification
c.706 + 629A > C	intron6	3.0% (1/33)	–	100% (1/1)	Uncertain significance (PM3, PP4_Moderate, PM2_Supporting)
c.707-59C > G	intron6	6.1% (2/33)	100% (2/2)	–	Likely pathogenic (PM3_Strong, PP4_Moderate, BS1)
c.1065 + 241C > A	intron10	33.3% (11/33)	81.8% (9/11)	18.2% (2/11)	Pathogenic (PM3_VeryStrong, PP4_Moderate, PM2_Supporting)
c.1199 + 502A > T	intron11	57.6% (19/33)	31.6% (6/19)	68.4% (13/19)	Pathogenic (PM3_VeryStrong, PP4_Moderate, PM2_Supporting)

The novel variant c.706 + 629A > C was identified in a patient with cPKU. After family verification, it formed a compound heterozygous with c.526C > T (p.Arg176Ter). This variant is not present in population databases (gnomAD no frequency). In silico analysis by RESCUE-ESE and ESEfinder, it predicts this variant is probably damaging to the protein structure. But these predictions have not been confirmed by functional studies. Therefore, it has been classified as a uncertain significance (PM3, PP4_Moderate, PM2_Supporting).

Combined with the phenotypic analysis, 68.4% of the PKU patients with c.1199 + 502A > T were associated with cPKU, while those with c.1065 + 241C > A preferred mPKU. The variant c.707-59C > G was identified in two patients with mPKU. According to ClinGen *PAH* Expert Panel Specifications for interpretation of genetic variants, these deep intronic variants were classified as likely pathogenic or pathogenic.

### Identification of the large-scale deletion/duplication of *PAH*

We selected five patients (in Table 3) and detected four kinds of deletion and duplication variants by MLPA, involving exons 4, 5, 6, 12, 1 and upstream (Additional file 1: Fig. S1). Full-length sequencing data showed the genomic regions of *PAH* gene deletions or duplicates in these samples. By comparison with the *PAH* reference sequence, we obtained the exons and introns contained in these regions. The results of analysis of the five samples were consistent with MLPA analysis. It shows that the single-gene full-length sequencing can analyze large-scale deletion/duplication within gene.

**Table 3:** Identification the large-scale deletion/duplication of *PAH*

Patient	Age	Pre-treatment Phe levels (μmol/L)	*PAH* allele 1		*PAH* allele 2
			MLPA result	Full-length sequencing result (involved exon and intron)	
38	1 m	2,254.80	exon4-5 deletion	NC_000012.11: g.103256126–103,272,397 del (exon4-5)	c.1162G > A(p.Val388Met)
39	1m3d	2484	exon6 deletion	NC_000012.11: g.103248768–103,249,219 del (exon6)	c.478C > T(p.Gln160Ter)
40	1y9m	1428	exon1 and upstream deletion	NC_000012.11: g.103311316-103315071del (exon1 and upstream)	c.1238G > C(p.Arg413Pro)
41	2m12d	768	exon1 and upstream deletion	NC_000012.11: g.103311023–103,312,086 del (exon1 and upstream)	c.158G > A(p.Arg53His)
42	1y	528	exon12 duplication	NC_000012.11:g.103233943_103235490dup (exon12)	c.721C>T(p.Arg241Cys)

### Distribution of *PAH* gene variant types

Through the supplementary detection of single-gene full-length sequencing, 683 of 687 PKU patients were completely genotyped. The variant types of the fully genotyped patients are summarized in Table 4. A total of 612 (89.6%) patients carried all variants in exons and flanking intron regions. We detected these variants by conventional sequence analysis, including Sanger sequencing and gene panels by NGS and whole-exome sequencing (WES). Thirty-three (4.8%) patients carried deep intronic variants that were identified by whole-genome sequencing (WGS) and single-gene full-length sequencing. In addition, 38 (5.6%) harbored the large-scale deletion/duplication that was detected by gene-targeted deletion/duplication analysis. Notably, among the molecular genetic testing used in PKU, only WGS and single-gene full-length sequencing detected all the above variants.

**Table 4:** Distribution of variant types and molecular genetic testing in PKU

Variant types	Proportion of Probands	Molecular Genetic Testing
SNV/Indel	94.4% (645/683)	Sequence analysis
In exons and flanking intron regions	89.6% (612/683)	sanger sequencing, gene panel, WES, WGS, single-gene full-length sequencing
In deep introns	4.8% (33/683)	WGS, single-gene full-length sequencing
*PAH* deletion/duplication	5.6% (38/683)	Gene-targeted deletion/duplication analysis: quantitative PCR, long-range PCR, MLPA, WGS, single-gene full-length sequencing

### Characteristics of variants in deep introns and untranslated regions

After our manual screening, there were seven nonbenign variants of deep introns in the PAHvdb database. Three of them were also identified in our study. In addition, in our study, we found a novel variant c.706 + 629A > C that had not been reported previously. Therefore, we analyzed a total of eight variants listed in the Additional file 1: Table S2.

**Fig. 2:**
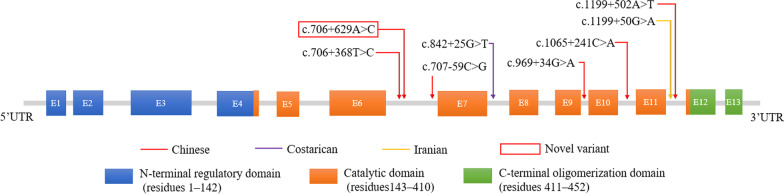
Analysis of 8 nonbenign *PAH* deep intronic variants: location and carrier population of the variants

Among these variants, seven were detected in Asian populations, including Chinese and Iranian populations. In terms of the location of the variants, all variants were located in the catalytic domain (Fig. 2).

## Discussion

PKU is a type of genetic disease with involvement of definite pathogenic genes and is treatable. Early, rapid and accurate genetic-based diagnosis is very important for subsequent patient treatment, genetic counseling and prenatal diagnosis. The previous diagnosis strategy suggested using sequencing analysis to detect variants in exons and flanking intron regions combined with MLPA to detect deletions and duplications [11].

In this study, we analyzed the genetic test results of 687 PKU patients and found that 5.4% of the patients could not be accurately diagnosed based on the previous strategy. Interestingly, full-length sequencing improved the diagnosis rate to more than 99% and can lead to rapid and efficient genetic-based diagnosis. To the best of our knowledge, this is the first time that single full-length sequencing of *PAH* has been used to identify PKU patients.

Full-length sequencing analysis suggested that deep intronic variants might be an important potential pathogenic mechanism of PKU. We found that nearly 90% of patients with previously undefined genotypes carried deep intron variants. Three deep intronic variants c.1199 + 502A > T, c.1065 + 241C > A and c.707-59C > G detected in our study were also reported in other variant spectrum studies of Chinese PKU patients [16, 17]. Our study provided additional patient data to support findings related to these three variants by sequencing a larger population. Notably, the c.1199 + 502A > T (2.8%, 19/687) and c.1065 + 241C > A (1.6%, 11/687) seemed to be found at a high frequency in our PKU patients. However, the pathogenicity of c.706 + 629A > C may need more experimental evidence or patient cases.

In the PAHvdb database, a total of 31 variants of deep introns are included. According to the previous criteria, we screened 8 nonbenign variants. Compared with other populations in the world, the frequency of deep intronic variants seems to be high in Asian, especially Chinese, PKU patients. We found that all of these deep intronic variants were distributed in the central domain, which includes Fe^3+^ ion binding sites involved in the binding of a cofactor [18]. The most commonly affected regions were introns 6 and 11. The distribution characteristics of deep intronic variants were consistent with the previous analysis of variants in the PAHvdb database and Chinese variant spectrum [11, 19].

Furthermore, we analyzed the genotypic-phenotypic correlation of these deep intronic variant carriers. Approximately 70% of patients with c.1199 + 502A > T had a severe cPKU phenotype. However, c.1065 + 241C > A and c.707-59C > G were associated with a lighter phenotype in PKU patients. Based on previous studies of genotype–phenotype correlation in PKU patients [16, 20], it is found that the variants of different positions will lead to different residual activities. Similarly, variations in different positions of the introns have different effects on protein expression. Martínez-Pizarro et al. [21] reported that c.1199 + 17G > A and c.1199 + 20G > C of *PAH* could cause a splicing defect by a novel mechanism involving U1snRNP binding downstream of the 5' splice site, resulting in exon 11 skipping and the formation of incomplete protein. Dericquebourg et al. [22] analyzed four hemophilia A patients with different clinical severity, who carried different deep intronic variants of *F8*. These variants lead to the creation of a de novo acceptor or donor splice site, and the formation of pseudoexon retention with different sequence lengths in the intron regions. For PKU patients, we speculate that the difference in phenotypic severity caused by deep intronic variants may be related to their location and impact on RNA splicing.

An increasing amount of studies have confirmed that deep intronic variants contribute to different diseases by affecting mRNA processing [23–25]. The splicing initiation of premRNA needs to be recognized by spliceosome and splice site (ss) sequences: the 5′ss (donor site), the 3′ss (acceptor site), and the branching point [26, 27]. Jin et al. [17] reported that the c.1199 + 502A > T variant of *PAH* acted to strengthen a cryptic branching point, and minigene expression showed that pseudoexon retention appeared in intron 11. The mechanism of pseudoexon inclusion caused by deep intronic variants may be due to activation of noncanonical splice sites, alterations to the splicing regulatory environment or loss of splice site competition [28, 29]. In addition, there are many other pathogenic mechanisms, such as disrupting transcription regulatory motifs or noncoding RNA [30]. Therefore, more experimental studies of the pathogenic mechanism of *PAH* deep intronic variants is required.

We analyzed data from 683 completely genotyped PKU patients, and deep intronic variants were carried in 4.8% of patients. Such variants cannot be detected by routine genetic analysis methods. WGS has been proven to be a powerful tool to identify causative variants residing outside coding regions in a variety of diseases [31, 32]. However, the cost of WGS sequencing is too high to be suitable for large-scale population screening. Similar to WGS, we designed a series of unique primers for *PAH* to amplify exons, deep introns and untranslated regions. And the time and cost of this single-gene full-length sequencing is similar to that of gene panel.

Moreover, our results indicated that the high-depth sequencing data obtained by full-length sequencing could also be used to analyze large-scale deletions/duplications within *PAH*. Therefore, this method of single-gene full-length sequencing not only has a lower detection cost than WGS but can also cover more regions of the targeted gene than routine genetic analysis methods. And using this method, we can find more deep intronic variants and micro-structure variants.

Even with single-gene full-length sequencing, four PKU patients (0.6%) did not have a clear genetic diagnosis. At present, phenylalanine metabolism may also be related to epigenetic factors [33]. Li et al. [34] reported that long noncoding RNAs (lncRNAs) were associated with PAH and modulated enzymatic activities by facilitating PAH-substrate and PAH-cofactor interactions. Therefore, epigenetic modification may be a new pathogenic mechanism of PKU.

## Conclusion

Our study offers proof that single-gene full-length sequencing can achieve rapid, efficient and cost-effective genetic-based diagnosis in PKU patients. We believe this sequencing technique can be implemented in medical practice to help with the diagnosis, genetic counseling, carrier screening and prenatal diagnosis of PKU patients.

## Supplementary Information

**Additional file 1.** **Table S1**: Other variants information of patients with unclear genotypes; **Figure S1**: MLPA results of patient 38-42; **Table S2**: Summary of nonbenign* PAH* variants in deep introns.

## Data Availability

The datasets used and/or analyzed during the current study are available from the corresponding author on reasonable request.

## Abbreviations

PKU: Phenylketonuria
Phe: Phenylalanine
PAH: Phenylalanine hydroxylase
mPKU: Mild PKU
cPKU: Classic PKU
ACMG: American College of Medical Genetics and Genomics
NGS: Next-generation sequencing
MLPA: Multiplex ligation-dependent probe amplification
WES: Whole-exome sequencing
WGS: Whole-genome sequencing
